# Exploring Chatgpt as a digital social support tool for bulimia nervosa

**DOI:** 10.1371/journal.pone.0345010

**Published:** 2026-03-26

**Authors:** Emre Vuraloğlu, Hatice Serra Malas, Elmas Merve Malas

**Affiliations:** 1 Department of Family Medicine, Kırşehir Training and Research Hospital, Kırşehir, Turkey; 2 Department of Psychiatry, Kırşehir Training and Research Hospital, Kırşehir, Turkey; 3 Department of Psychology, Konya Food and Agriculture University, Konya, Turkey; University of Toronto, CANADA

## Abstract

Social support is essential in eating disorder care. Beyond traditional sources, ChatGPT offers a novel and underexplored digital approach that may complement but also potentially challenge existing support systems during treatment and recovery. This study aims to evaluate the potential of ChatGPT as a digital social support tool for individuals with bulimia nervosa. This qualitative and descriptive study used 32 bulimia-related scenarios based on the Edinburgh Bulimia Test. ChatGPT’s responses were evaluated via structured content analysis using a scenario-based approach. Each response was independently rated by a psychiatrist and a clinical psychologist across four social support categories: emotional, informational, appraisal, and instrumental. Ratings were made using a 5-point Likert scale (1 = very poor, 5 = excellent). Inter-rater reliability was assessed using Cohen’s Kappa coefficients. Friedman and Mann–Whitney U tests were applied to compare support types. Cohen’s Kappa indicated agreement between raters (κ = 0.58–1.00). ChatGPT demonstrated the highest performance in the informational support category across all scenarios, receiving the highest possible scores from both raters. The Friedman test revealed statistically significant differences among the support categories (p < 0.001). Post-hoc pairwise comparisons showed that informational support was rated significantly higher than all other support types (p < 0.001), while instrumental support received the lowest ratings. No statistically significant difference was observed between emotional and appraisal support categories (p = 1.000). This study highlights ChatGPT’s emerging potential as a digital provider of social support in bulimia nervosa, particularly in delivering informational support.

## Introduction

Bulimia Nervosa (BN) is a serious psychiatric disorder characterized by recurrent episodes of binge eating followed by compensatory behaviors, significantly impairing quality of life [1,2]. Individuals with BN often exhibit an intense fear of weight gain and distorted body image perceptions [1,2]. The condition is associated with a lifetime prevalence of depression as high as 76.3%, as well as increased anxiety, heightened impulsivity, and a markedly elevated risk of suicide attempts, contributing significantly to overall mortality and suicide risk [3–5]. Feelings of shame, guilt, and secrecy frequently accompany the disorder, leading to delays in help-seeking behaviors and reduced access to professional care [6–8].

Social support plays a crucial role in the treatment trajectory of BN [9]. A nonjudgmental and empathetic attitude from close contacts, such as family members, partners, or friends, not only enhances motivation to seek professional help but may also reduce the risk of relapse [10,11]. However, such support is not always available, especially for individuals who experience social isolation or stigma, leading to unmet needs in emotional care. In response, digital health tools have emerged as scalable alternatives for bridging these gaps.

Recently, artificial intelligence (AI), such as ChatGPT (Chat Generative Pre-trained Transformer), has been explored for its potential to simulate social support through empathetic responses, judgment-free communication, and 24/7 availability [12,13]. It has also been shown to serve as a supportive tool for patient communication without replacing clinical judgment or medical guidance [14]. Despite this promise, the effectiveness and safety of these systems, specifically in populations with BN, remain largely unexplored. Therefore, this study focuses on Bulimia Nervosa, a complex psychiatric disorder in which stigma, guilt, secrecy, and high rates of comorbid depression, anxiety, and suicidality collectively restrict help-seeking behaviors and access to social support.

This study aims to investigate the potential of ChatGPT as a digital social support provider for individuals with Bulimia Nervosa. By doing so, this exploratory study seeks to contribute novel insights to the growing literature on AI-driven interventions in the context of eating disorders.

## Materials and methods

### Study design

This qualitative and descriptive study aimed to evaluate the social support function of ChatGPT, a large-language-model-based artificial intelligence system, in response to self-reported expressions of individuals with bulimia nervosa. We conducted the study using scenario-based expressions developed from the Edinburgh Bulimia Investigation Test (EBIT). We did not collect any real patient data; instead, standardized symptom narratives were generated from validated diagnostic items to ensure ethical rigor, replicability, and control over input variability. This design allowed the study to isolate ChatGPT’s linguistic and empathic capacities without the confounding influence of patient heterogeneity.

### Data source

The data source was the EBIT, originally developed by Henderson and Freeman (1987) [15]. We specifically selected the EBIT because it consists of behaviorally specific, clinically grounded items that mirror real-world experiences of bulimic patients. This made it ideal for constructing authentic, scenario-based prompts for the AI model. The test consists of 33 items and includes a total of 36 questions when multi-part items are considered. In this study, We excluded question 31 due to its structural and semantic similarity to question 30, resulting in a final set of 32 questions. We presented all questions to ChatGPT in Turkish. An example question and ChatGPT’s response are presented in Table 1.

**Table 1:** Sample patient scenario and ChatGPT-4o response.

Question:	
Hello. I am a 35-year-old woman with bulimia nervosa. I have no other illnesses or medications. I do not have a regular daily meal schedule.	
**ChatGPT Response:**	
Hello, thank you for your message. Coping with bulimia nervosa (BN) can be challenging but establishing a regular meal plan is an important part of this process. I would like to share some basic information and suggestions that may help you. However, please remember that the best support comes from working with a psychiatrist, psychologist, and a nutrition specialist (dietitian).	
Why Are Bulimia Nervosa and Regular Eating Important?	
• Irregular eating can lead to fluctuations in blood sugar levels, sudden binge eating episodes, and subsequently to compensatory behaviors such as vomiting. • Eating regularly supports both physical and emotional balance and facilitates recovery.	
A Simple Daily Meal Plan Suggestion to Start With	
This plan is an average starting point. It is important to personalize it together with a professional based on your individual needs.	
**Mea**	**Example**
Breakfast (08:00–09:00)	1 slice whole wheat bread + 1 egg + a few olives + tomato/cucumber + 1 glasses of milk
Mid-Morning Snack (10:30–11:00)	1 piece of fruit (e.g., apple or pear)
Lunch (12:30–13:30)	Grilled chicken or lentil stew + bulgur pilaf + yogurt + salad
Afternoon Snack (15:30–16:00)	A handful of walnuts or almonds + ayran
Dinner (18:30–19:30)	Vegetable dish + yogurt + whole grain bread
Optional Late-Night Snack (21:00)	Warm milk or a piece of fruit
Behavioral Recommendations:	
• Try not to skip meals; skipped meals can more easily trigger binge eating episodes. • Try to eat slowly and mindfully, rather than quickly. • Try to be compassionate and supportive toward yourself, rather than judgmental. • When you experience a binge episode, try to understand the reason without feeling guilty. This can positively contribute to your recovery.	
Getting Help Is Important:	
• BN is a condition that cannot be treated by willpower alone; it can improve with professional support. • Psychotherapy (especially Cognitive Behavioral Therapy) is effective in the treatment of BN. • A psychiatrist can plan pharmacological treatment if necessary.	
If you wish, I can assist you further by providing a personalized meal plan or additional supportive resources. I am here for you.	

Each of the 32 questions was transformed into a symptom-based narrative to form clinical scenarios. In July 2025, each of the 32 scenario-based questions, derived from the Turkish-validated and reliability-tested form of the Edinburgh Bulimia Investigation Test (EBIT), was submitted in Turkish to the most recent version of ChatGPT (GPT-4o) on two separate days. The responses generated were recorded, and content analysis revealed that the outputs were nearly identical across both sessions, demonstrating a high level of consistency in the model’s responses. We recorded the model’s responses exactly as generated, without any modifications.

### Evaluation process and statistical analysis

The responses generated by ChatGPT-4o were analyzed according to the four primary types of social support defined by House: emotional, informational, appraisal, and instrumental support [16]. One psychiatrist and one clinical psychologist independently evaluated each response, both with over five years of experience and currently working in advanced tertiary-care hospitals. Despite the limited number of raters, the inclusion of both a psychiatrist and a clinical psychologist allowed for complementary and multidimensional evaluation of the AI’s responses. Considering the heavy clinical workload and limited availability of mental health professionals in Turkey, expanding the rater pool was not practically feasible but did not compromise interpretive depth or analytical validity. A five-point Likert scale was used (1 = poor, 5 = excellent).

Descriptive statistics were calculated for each support domain. We assessed inter-rater agreement using Cohen’s Kappa coefficients. Differences in ChatGPT’s performance across the four social support types were evaluated using the Friedman test. Post-hoc pairwise comparisons were conducted using the Bonferroni-corrected Mann–Whitney U test. Effect sizes were calculated for both Friedman and Mann–Whitney U tests to determine the magnitude of observed differences. All statistical analyses were performed using IBM SPSS Statistics version 27, with a significance level set at p < 0.05.

### Ethical considerations

No data were collected directly from human participants in this study. Only content generated by an artificial intelligence model was evaluated. Therefore, formal ethical approval was not required. Two expert raters (one psychiatrist and one clinical psychologist) participated only as evaluators of the AI-generated texts. Written informed consent was obtained from both expert raters prior to participation.

## Results

The responses generated by ChatGPT-4o were evaluated across four categories of social support: emotional, informational, appraisal, and instrumental. Inter-rater agreement between the psychiatrist and psychologist was moderate for emotional (κ = 0.60) and appraisal support (κ = 0.58), perfect for instrumental support (κ = 1.00), and uncomputable for informational support due to no variability in scores. In fact, both raters assigned the maximum possible scores with perfect agreement across all scenarios; however, due to the absence of score variability, Cohen’s Kappa for informational support could not be computed for statistical reasons.. Descriptive statistics showed that the highest mean score was observed for informational support, followed by appraisal, emotional, and instrumental support (Table 2).

**Table 2:** Descriptive statistics for ChatGPT-4o responses across four types of social support.

Social Support Type	Mean (Standard Deviation)
Emotional	4.28 (0.73)
Informational	5.00 (0.00)
Appraisal	4.31 (0.64)
Instrumental	1.97 (0.31)

Instrumental support refers to tangible forms of assistance, including financial aid and access to necessary services. The goal of such support is to help individuals resolve their problems and reduce associated stress. Additionally, providing temporal support may allow individuals to allocate more time for themselves, thereby contributing to stress reduction. In the present study, instrumental support in the responses generated by ChatGPT was identified only in the first scenario, in which the suggestion of a daily meal plan was interpreted by the evaluators as a form of tangible assistance. The evaluators did not observe any clear instrumental support in the remaining scenarios. However, all scenarios included a substantial amount of informational support, with ChatGPT offering detailed advice, behavioral strategies, and suggestions regarding potential actions. ChatGPT consistently received full scores from both evaluators in the domain of informational support, as its responses aligned with the definitions outlined in the Diagnostic and Statistical Manual of Mental Disorders, Fifth Edition (DSM-5), published by the American Psychiatric Association (2013), particularly within the category of “Feeding and Eating Disorders.” Emotional support was defined as expressions of love, respect, empathy, and compassionate behavior toward the individual. Upon closer examination of the scoring differences between raters, it was noted that the clinical psychologist tended to rate alternative cognitive statements as strong emotional support, whereas the psychiatrist evaluated these statements as partial emotional support. Regarding appraisal support which refers to the provision of feedback, affirmation, and comparative information that enables self-evaluation the inter-rater agreement was similarly moderate.

As shown in Table 3, a Friedman test revealed a statistically significant difference in ChatGPT’s performance across the four types of social support (χ² = 78.52, p < 0.001). Post-hoc pairwise comparisons using the Bonferroni-corrected Mann–Whitney U test indicated that all comparisons were statistically significant except for the comparison between emotional and appraisal support (p = 1.000). The effect size for the Friedman test was large (η² = 0.75), and pairwise Mann–Whitney comparisons yielded large effects (r > 0.50) for all significant contrasts. These large effect sizes indicate that the differences between social support types generated by ChatGPT are substantial in magnitude rather than trivial.

**Table 3:** Results of Friedman test and Bonferroni-adjusted Mann–Whitney U post-hoc comparisons across social support types.

Analysis	Comparison Social Support Types	U/ χ²	p-value	Significant (Bonferroni α = 0.0083)
Friedman Test	–	78.52	p < 0.001	–
Post-hoc Test	Emotional vs Informational	192.0	p < 0.001	Yes
Post-hoc Test	Emotional vs Appraisal	511.5	p = 1.000	No
Post-hoc Test	Emotional vs Instrumental	1021.5	p < 0.001	Yes
Post-hoc Test	Informational vs Appraisal	848.0	p < 0.001	Yes
Post-hoc Test	Informational vs Instrumental	1024.0	p < 0.001	Yes
Post-hoc Test	Appraisal vs Instrumental	1022.5	p < 0.001	Yes

## Discussion

This study provides empirical evidence that ChatGPT may serve as a complementary digital tool for delivering social support to individuals with bulimia nervosa, particularly in informational domains. The model received high ratings from expert evaluators in the informational support category, indicating a strong capacity to provide accurate and relevant content. In contrast, its performance in delivering instrumental support was notably limited. Emotional and appraisal support were rated at moderate levels, with no statistically significant difference observed between the two.

Social support is a key factor influencing the clinical course of eating disorders. In one of the studies that quantitatively demonstrated this relationship, Stern et al. (2023) reported a bidirectional association between perceived social support and eating disorder symptoms [17]. This prospective study demonstrated that low levels of social support may contribute to the worsening of eating disorder symptoms. In contrast, the presence of such symptoms over time may negatively influence the perception of available support [17]. These findings underscore the dynamic and reciprocal nature of support systems in the context of eating disorders.

In a two-armed randomized controlled trial conducted by Sharp et al. (2025), the effectiveness of a digital chatbot developed for the prevention of eating disorders was evaluated. The findings demonstrated that the chatbot led to improvements in eating disorder pathology, psychosocial impairment, depression, and anxiety [12]. Participants described the chatbot as an accessible, practical, scalable, informative, and supportive tool for early intervention [12].These results suggest that artificial intelligence–based tools designed to provide social support may play a preventive and supportive role in managing eating disorders. The strong performance of ChatGPT in providing informational support in the present study is consistent with these findings. In their 2024 study, Lee and Hahn examined the relationship between mind perception and the ability of chatbots to provide social support [18]. They reported a significant association between higher levels of mind perception and the perceived capacity of chatbots to deliver social support. However, participants noted that while chatbots were effective in delivering informational support, they were insufficient in providing emotional support [18]. A similar finding was observed in the present study.

For social support to be effective, both the objective presence of the support provider and the psychological structure of the recipient are important [19]. Individuals with low self-esteem are more likely to perceive technology in a negative light [20]. A person’s psychological structure is closely related to their self-perception. According to social comparison theory, individuals evaluate their abilities, opinions, and emotions by comparing themselves with others [21]. Likewise, individuals compare ChatGPT responses with their own values and internal belief systems [22]. Among the key factors determining the acceptance of ChatGPT in the healthcare field are users’ perceptions of competence, reliability, and trustworthiness. In a large-scale cross-sectional study demonstrated that the factors most strongly influencing trust in ChatGPT during healthcare-related decision-making processes were perceived competence and reliability [23].It has been acknowledged that chatbots, including ChatGPT, may occasionally produce misleading or false information [24]. When individuals with low self-esteem encounter such responses, their levels of stress and anxiety may increase [22]. The impact of inaccurate information generated by chatbots on users’ perceptions and trust in these tools has not been sufficiently explored [24].

Chatbots may be misused if users are not adequately trained [25]. Although informative, chatbots may misinterpret unforeseen user queries and provide inappropriate responses [26,27]. While chatbots can be effective for delivering information and engaging in simple conversations, their limitations in accurately understanding users mean that temporary solutions may be insufficient to eliminate risks [26]. Moreover, although personalized responses may facilitate treatment processes, they also raise significant concerns regarding the protection of personal data [28]. Chatbots operate on existing databases; however, encoding human cognitive and emotional structures in a reductionist manner can lead to stereotypical responses. Therefore, facilitating personalized treatments through strong data governance measures to safeguard sensitive information, along with adopting a collaborative approach, offers a comprehensive pathway to empower both patients and healthcare professionals.

In an observational study conducted by Chan et al. (2022), the technical and content-related challenges of designing a chatbot for preventing eating disorders were discussed [26]. The researchers emphasized that, for such systems to be effective, it is not sufficient to provide information alone; they must also be capable of delivering user-specific responses, employing empathetic language, and offering behavioral guidance [26]. In the present study, ChatGPT demonstrated strong performance in delivering informational support but showed limited capacity in providing instrumental support. These findings are consistent with the literature and highlight that achieving a balanced provision of different types of social support via AI systems remains an area in need of further development. From another perspective, the study also showed that ChatGPT’s responses to the presented scenarios provided individuals with essential information and suggestions that could help them manage their difficulties. Such responses may contribute to greater awareness and understanding of their condition, enabling individuals to better identify, analyze, and cope with their problems. This, in turn, may promote insight into the illness and reinforce the perception that the situation is treatable, potentially reducing delays in seeking professional help.

In a study conducted by Schnepper et al. (2024), it was shown that large language models (LLMs) may reflect gender- and sexual-orientation–based biases in their responses to eating disorder scenarios [29]. The researchers found that gender and sexual orientation variables influenced both the treatment recommendations and the language used by the model in cases of anorexia nervosa and bulimia nervosa. This highlights the need for careful evaluation of neutrality and inclusivity when implementing LLM-based systems in mental health contexts. Although gender and sexual orientation were not manipulated in the present study, consistency in ChatGPT’s delivery of different types of social support was examined, and limitations in certain domains were identified. As emphasized by Schnepper et al., it is essential to systematically assess such systems for ethical, cultural, and social biases, particularly in sensitive areas such as mental health.

This study examines the use of ChatGPT as a digital social support tool in bulimia nervosa, a clinically sensitive mental health disorder associated with an increased risk of suicide and serious medical complications [30]. In such clinically sensitive contexts, the use of artificial intelligence–based systems raises important ethical concerns, including the potential generation of inaccurate or incomplete information, inappropriate reassurance, delays in seeking professional help, and excessive reliance on non-human sources of support. Despite these concerns, current qualitative evidence suggests that LLMs used in mental health–related ethical contexts may hold promise as emotionally attuned and context-sensitive companions in ethical decision-making processes [31]. However, rather than replacing clinical judgment, these tools may serve to alleviate emotional burden, enhance therapeutic reflection, and promote ethically sound care in complex, high-pressure situations. For these reasons, when the use of chatbots in mental health–related contexts is evaluated, it is of critical importance to clearly define usage boundaries, explicitly state system limitations, and maintain continuous human oversight. While chatbots may function as practical and supportive tools, they cannot replace clinicians in terms of human relational skills, ethical responsibility, and therapeutic decision-making.

This study has several notable strengths. The use of a scenario-based qualitative evaluation framework grounded in a validated instrument (EBIT) and the involvement of two independent expert raters from psychiatry and clinical psychology enhance both the reliability and depth of the analysis. The systematic classification of ChatGPT’s responses into established social support domains further contributes to the methodological rigor and conceptual clarity of the study. However, several limitations should be acknowledged. The use of questions and responses in Turkish may limit the generalizability of the findings to other languages and cultural contexts. Therefore, replication in other languages and cultural contexts is necessary before broader generalization. Moreover, while expert-based evaluations are valuable, they inherently involve a degree of subjectivity, and the limited number of raters (two experts) should also be considered as a potential limitation affecting the generalizability of the findings. One of the main limitations of our study is that chatbot responses were evaluated using predefined and standardized prompts. The absence of real patient inputs indicates that this study should be regarded as an exploratory, proof-of-concept investigation rather than as evidence for real-world applicability. The use of prompts written by real individuals, which may better reflect real-life usage patterns, could represent an important area for future research. Individualized assessment of the disorder is crucial for effective treatment planning and management. However, focusing solely on the presenting symptoms may lead to neglecting environmental factors and difficulties in other life domains. Such an approach carries the risk of overlooking comorbid conditions, which may adversely affect the treatment process and potentially cause harm to the individual. Another limitation of our study is that it did not evaluate crisis or emergency responses. As is well known, bulimia nervosa is a disorder associated with an increased risk of suicide and serious medical complications. Future studies should systematically assess the capacity of AI-based systems to recognize crisis situations, generate safe responses, and provide appropriate emergency referrals. Finally, although purposively selected, the relatively small sample of scenarios may constrain the broader generalizability of the results.

## Conclusion

This study demonstrates that ChatGPT consistently delivers a high level of informational social support in response to scenarios related to bulimia nervosa. However, its capacity to provide instrumental support remains limited. While the findings suggest that ChatGPT may serve as a reliable source of accurate and evidence-based information, they also highlight the need to improve its ability to offer directive guidance, particularly within sensitive clinical domains such as eating disorders. Given the frequent comorbidities associated with bulimia nervosa, as well as its elevated risks of mortality and suicide, the clinical approach to this patient population requires sensitivity. Concerns about stigma and the tendency to avoid treatment-seeking may be partially alleviated by support received from chatbots, potentially serving as a first step toward professional help. Therefore, whether delivered online or in person, social support interventions are expected to enhance accessibility to early intervention, strengthen motivation for change, and increase the likelihood of treatment-seeking behavior. These findings should be interpreted with caution, as this study provides preliminary insights based on standardized scenarios rather than real-world user interactions. Accordingly, the results should be regarded not as evidence for real-world clinical applications, but as exploratory and proof-of-concept findings.
